# MicroRNA and circRNA Expression Analysis in a Zbtb1 Gene Knockout Monoclonal EL4 Cell Line

**DOI:** 10.3389/fcimb.2021.706919

**Published:** 2021-07-05

**Authors:** Jun-Hong Wang, Chun-Wei Shi, Yi-Yuan Lu, Yan Zeng, Ming-Yang Cheng, Ru-Yu Wang, Yu Sun, Yan-Long Jiang, Wen-Tao Yang, Dan-Dan Zhao, Hai-Bin Huang, Li-Ping Ye, Xin Cao, Gui-Lian Yang, Chun-Feng Wang

**Affiliations:** ^1^ College of Veterinary Medicine, Jilin Agricultural University, Changchun, China; ^2^ Jilin Provincial Engineering Research Center of Animal Probiotics, Jilin Agricultural University, Changchun, China; ^3^ Key Laboratory of Animal Production and Product Quality Safety of Ministry of Education, Jilin Agricultural University, Changchun, China

**Keywords:** Zbtb1, EL4, microRNA, circRNA, RNA-seq

## Abstract

Zinc finger and BTB domain containing 1(Zbtb1) is a transcriptional suppressor protein, and a member of the mammalian Zbtb gene family. Previous studies have shown that Zbtb1 is essential for T-cell development. However, the role of Zbtb1 in T-cell lymphoma is undetermined. In this study, an EL4 cell line with Zbtb1 deletion was constructed using the CRISPR-Cas9 technique. The expression profiles of microRNA and circRNA produced by the control and gene deletion groups were determined by RNA-seq. In general, 24 differentially expressed microRNA and 16 differentially expressed circRNA were found between normal group and gene deletion group. Through further analysis of differentially expressed genes, GO term histogram and KEGG scatter plot were drawn, and three pairs of miRNA and circRNA regulatory relationships were found. This study describes the differentially expressed microRNA and circRNA in normal and Zbtb1-deficient EL4 cell lines, thus providing potential targets for drug development and clinical treatment of T-cell lymphoma.

## Introduction

Expression of Zbtb1 is necessary for normal lymphoid development. Zbtb1 maintains genomic integrity in immune progenitors during the process of replication and differentiation, while Zbtb1 deficiency increases DNA damage and p53-mediated apoptosis, mRNA encoding Zbtb1 is most highly expressed in hematopoietic stem cells, thymocytes and pre-B cells, In addition to its role in T cell development, it was also demonstrated to be involved in the differentiation of B cells and NK cells (Siggs et al., 2012; Punwani et al., 2012; Cao et al., 2016; Cao et al., 2018). In particular, it is very important for the development of NKp46^+^ ILC3 cells (Lu et al., 2017). Loss of Zbtb1 makes therapy-resistant T cell leukemia cells sensitive to L-asparaginase, revealing that Zbtb1 may be a key regulator of the nutritional stress response (Williams et al., 2020). Some studies have shown that Zbtb1 is a tumor suppressor in breast cancer cells. MiR-23b-3p inhibits breast cancer cell proliferation and tumor growth by targeting Zbtb1 to regulate aerobic glycolysis in tamoxifen-resistant cells (Kim et al., 2014).

MicroRNAs (miRNAs), a kind of endogenous noncoding RNA with regulatory function in eukaryotes, are approximately 20 to 25 nucleotides in length. Recent studies have shown that miRNA is involved in a variety of regulatory pathways, including development, viral defense, hematopoietic process, organogenesis, cell proliferation and apoptosis, fat metabolism and so on. In recent years, thousands of circular RNA molecules have been found in large numbers *in vivo*, enabling a new and in-depth understanding of this kind of “dark matter”. The primary functions of circular RNA molecules are (1) acts as miRNA sponge (ceRNA); (2) regulates gene transcription; (3) regulates RNA binding proteins; (4) participates in protein translation (Zhang P. et al., 2020). In addition, because cyclic RNA is insensitive to nuclease and is more stable than linear RNA, circRNA has the potential to become a new diagnostic marker. At present, the microRNA and circRNA related to zbtb1 protein in T lymphomas have not been studied.

CRISPR (clustered regularly interspaced short palindromic repeats) is an immune mechanism from bacteria that degrades invading viral DNA or other exogenous DNA. Cas9 first binds to crRNA and tracrRNA to form a complex and then binds to the adjacent motif (protospacer adjacent motifs, PAM) of the anterior interregion sequence and invades DNA to form a RNA-DNA complex structure that then cleaves the target DNA double strand to cause a DNA double strand break (Zhong et al., 2018). So far, the CRISPR/Cas9 system has been widely used in genomic modification of animals, plants and microorganisms.

Several studies show a contribution of these so-called competing-endogenous RNA networks in various cancer entities (Doudna and Charpentier, 2014). It is of great significance to study the expression of microRNA and circRNA in T-cell lymphomas and the relationship between mRNA and expression.

## Materials and Methods

### Cell Culture

EL4 cells were cultured in DMEM (Gibco) with 10% fetal bovine serum (Gemini) in a 37°C incubator providing 5% CO_2_.

### Construction of an EL4 Cell Line With Zbtb1 Gene Knockout by the CRISPR-Cas9 Technique

PCR products were sequenced with EL4 cell genome as a template, and the sequencing results were compared to the target sequence on NCBI on Ape software. Within the normal range of Sanger sequencing. sgRNA is an important part of the CRISPR gene knockout system. It was previously found that guide RNA consists of two parts-tracRNA and crRNA, fusion expression, that is, sgRNA can also perform the function of guide and bind to the Cas9 protein, leading the Cas9 enzyme to target genomic DNA for splicing. First, the sgRNA was designed, and synthesis of the sgRNA scaffold was completed. Then, the corresponding cell groups of sgRNA with the highest editing efficiency were digested into single cells with trypsin and counted. Single cells were divided into 96-well plates and cultured in incubator for 3-5 days. Surviving monoclonal cells were observed under a microscope. The growing monoclonal cells were expanded and cultured, and the genomic DNA was extracted. After the target fragment was amplified and verified by Sanger sequencing, double-knock clones were identified (that is, the mutations of the two chromosomes were not multiples of 3).

### Comparison of the Growth Rate of Subcutaneously Transplanted Tumors Derived From Different EL4 Cell Lines in C57BL/6 Mice

To evaluate differences in the growth rate of subcutaneously transplanted tumors, wild type EL4 cells and Zbtb1 gene knockout EL4 cells were grown in C57BL/6 mice with an intact immune system to evaluate the role of the Zbtb1 gene in the occurrence and development of T lymphocyte tumors. C57BL/6 mice were randomly divided into two groups with 8 mice in each group. Wild type EL4 cells and Zbtb1 knockout EL4 cells were inoculated with 1 × 10^6^ cells per mouse, respectively. After cell inoculation, tumor volume and the body weight of mice were measured twice a week until the average volume of transplanted tumor in nude mice was greater than 2000 mm^3^. At the end of the formal experiment, mice were euthanized by carbon dioxide asphyxiation.

### MicroRNA Bioinformatics Analysis

Known miRNAs were annotated by comparing the sequenced reads with known miRNAs in the miRBase v20 database. At the same time, the sequence was compared to the Rfam database to analyze ncRNA distribution in smallRNA. The sequence was compared to the whole genome sequence of the species, and the new miRNA was predicted using a folding model. The differential miRNA expression, clustering pattern analysis and target gene function prediction among different samples were analyzed.

### MicroRNA and Its Target Gene Prediction

First, reads obtained by sequencing are aligned to the reference genome, regardless of the reads, for multiple sites. Then, miRDeep2 uses the alignment information of reads on the reference genome to calculate the secondary structure of each possible microRNA precursor and evaluate it. According to the structure and score of these precursors, new microRNA sequences can be predicted.

### Differential microRNA Expression Analysis

Some microRNAs are distributed in clusters in the genome, and these microRNAs are transcribed synchronously. For samples with biological repetition, a microRNA difference analysis was performed using DESeq2 (V1.6.3) in the Bioconductor software package. In special cases, gene difference analysis was performed using edgeR (V3.4.6) in the Bioconductor software package. The software used in this analysis was DESeq2.

### CircRNA Identification and Bioinformatics Analysis

The most important principle of predicting circRNA by high-throughput sequencing technology is to identify the reverse splicing sequence, namely, back-splicing reads. Based on the.sam file of the comparison result for each sample, CIRI (V2.0) software was used to predict the position information before and after the formation of circRNA. The CIRI software uses the CIGAR value in the SAM format to analyze and scan the PCC signal (paired chiastic clipping signals) from the.sam file. The experimental process of cyclic RNA sequencing includes annular RNA extraction, annular RNA sample quality detection, library construction, library purification, library detection, library quantification, generation of sequencing clusters and computer sequencing. The different libraries were mixed according to the effective concentration and the target amount of data from the machine and were then sequenced on the Illumina platform.

### CircRNA Annotation and Its Expression Analysis

circBase is a database built by collecting and integrating published circRNA data. By comparing the location information of all predicted circRNAs with the known circRNA in the circBase database, the newly predicted novel circRNA and the known circRNA can be distinguished. At present, for the vast majority of circRNAs, the complete sequence cannot be obtained, so we can only use the junction reads at the back-splicing site of circRNA to calculate its expression, and SRPBM (spliced reads per billion mapping) was used to normalize reads.

### Cluster Analysis of Differentially Expressed Genes

Cluster analysis was performed to calculate the similarity of the data and classify the data according to the similarity, so that the microRNA (circRNA) with the same function or close relationship can be clustered into classes to identify unknown functions or known unknown functions and infer whether they participate in the same metabolic process or cell pathway together. The TPM (Transcripts per million, TPM=RNA reads number x10^6^/total reads) value of different microRNAs (circRNA) under different experimental conditions as the expression level was used to perform hierarchical clustering analysis. The primary feature of this method is its ease of use for generating trees.

### GO Enrichment Analysis of Differentially Expressed Target Genes

This analysis first maps all differentially expressed microRNA target genes and circRNA source genes to each term in the Gene Ontology database, calculates the number of genes per term, and then uses hypergeometric tests to identify the GO items that are significantly enriched in differentially expressed microRNA target genes and circRNA source genes compared to the whole genome background. After the calculated p-value is corrected by Bonferroni, the threshold is corrected p-value ≤ 0.05. After being corrected by Bonferroni, the calculated p-value is used as a threshold. The GO term that meets this condition is defined as the GO term that is significantly enriched in the differentially expressed target genes.

### KEGG Enrichment Analysis of Differentially Expressed Target Genes

*In vivo*, different genes coordinate with each other to exercise their biological functions, and the most important biochemical metabolic pathways and signal transduction pathways involved in differentially expressed microRNA (circRNA) target genes can be determined by analysis of significantly enriched pathways. KEGG (Kyoto Encyclopedia of Genes and Genomes) is the primary public database used to identify pathways. Significantly enriched pathway analysis was based on the KEGG pathways, and the hypergeometric test was used to determine whether each pathway was significantly enriched in differentially expressed microRNAs (circRNAs) compared with the whole genomic background.

### Statistical Analysis

All results were expressed as the mean ± standard deviation (SD) of three independent experiments. Statistical analysis was performed using t-test. All statistical tests were indicated by two tails, P<0.05 was considered significant.

## Results

### Constructing EL4 Cells With Zbtb1 Gene Knockout

#### sgRNA Screening *In Vitro*

**Figure 1:**
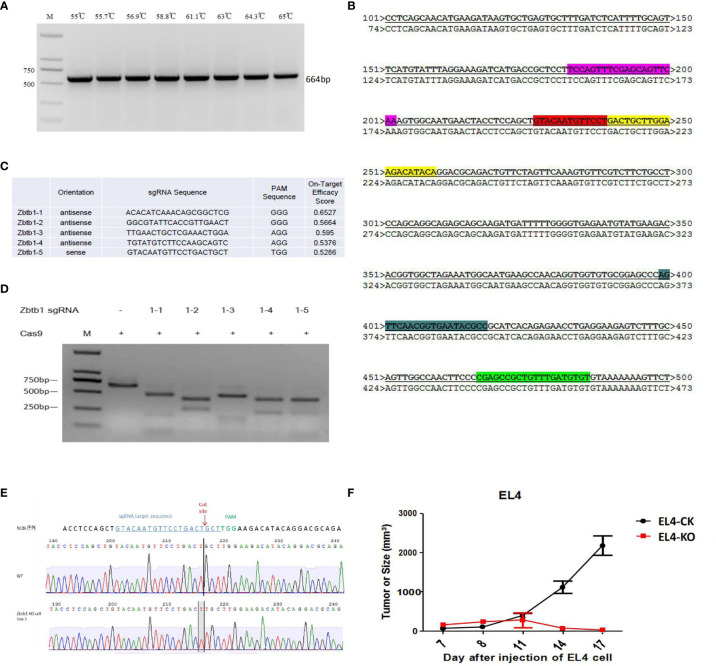
**(A)** The target fragment of the EL4 cell genome was amplified by PCR; **(B)** The result of sequencing compared to the target sequence on NCBI. The highlighted part is the designed sgRNA sequence; **(C)** The design of 5 sgRNA sequences; **(D)** Zbtb1 sgRNA correctly mediates the cleavage function of Cas9 protein to the target fragment *in vitro*; **(E)** Zbtb1 KO-EL4 cell lines were sequenced; **(F)** Comparison of growth rate of subcutaneously transplanted tumor in C57BL/6 mice.

The target fragment of the EL4 cell genome was amplified by PCR (Zbfb1_survy_F: ACGACATCTACTTCCAAGCACACA; Zbfb1_survy_R: GGTAAGCTCTTGTCGTGTTTTGGT), and the length of the fragment was 664 bp (**Figure 1A**), the target sequence was consistent with the NCBI sequence and the alignment results (**Figure 1B**). Design of the sgRNA(small guide RNA) target sequence targeting the Zbtb1 gene was then performed (**Figure 1C**, X represents the designed sgRNA sequence). Cas9 protein and sgRNA were incubated with the target fragment at 37°C for 2-3 hours, and then analyzed by agarose gel electrophoresis. Results are shown in **Figure 1D**. All five sgRNAs correctly mediated cleavage of the target fragment by Cas9 protein, and gray band analysis showed that the gene editing ability of sgRNA1-5 was better than that of other sgRNAs. Therefore, sgRNA1-5 was selected for the follow-up experiment.

#### Culture and Screening of Monoclonal Cells

A total of 62 clones survived and were identified according to the speed of monoclonal growth. When the gene double-knock monoclonal identification was successful, the monoclonal screening and identification was stopped. Results of sequencing the knockout sites of monoclonal cells showed that monoclonal 1, monoclonal 2, monoclonal 6 and monoclonal 7 were monoclonal cells belonging to the allelic double knock and monoclonal 1 sequencing map analysis, such as (**Figure 1E**). The Zbtb1 KO-EL4 cell line sequencing analysis showed that a base T was inserted at the sgRNA cleavage site. The two alleles of monoclonal 1 were inserted into a base T, and the frameshift mutation resulted in knockout of the Zbtb1 gene. This monoclonal is a double knock cell line of the Zbtb1 gene.

#### Comparison of the Growth Rate of Subcutaneously Transplanted Tumor in C57BL/6 Mice With Different EL4 Cell Lines

The subcutaneous growth rate of EL4 cells with Zbtb1 knockout was significantly slower than wild type EL4 cells in C57BL/6 mice (**Figure 1F**).

### Forecast of microRNA and Statistics of Annotated Results

**Figure 2:**
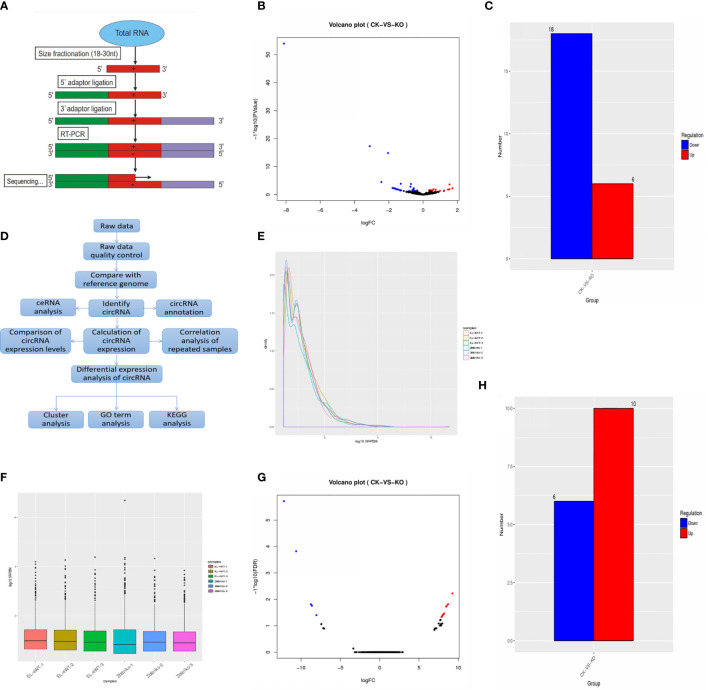
**(A)** MicroRNA sequencing process; **(B)** Differential expression of microRNAs. Significantly different microRNAs with red dots are upregulated, and blue dots are downregulated. Abscissa represents multiple changes in microRNA expression in different samples, and ordinate represents statistically significant differences in microRNA expression; **(C)** Sample differences compare up- and downregulated microRNAs; **(D)** CircRNA analysis process; **(E)** SRPBM distribution map. Abscissa is log10 (SRPBM), and vertical coordinate is circRNA density; **(F)** SRPBM box chart. Abscissa is the name of the sample, and ordinate is the log10 (SRPBM). Box chart for each region of the five statistical quantities (top to bottom is the maximum, upper quartile, median, lower quartile and minimum); **(G)** In the differential circRNA expression map, the red dots represent upregulated circRNAs, and the blue dots represent downregulated circRNAs. Abscissa represents multiple changes in circRNA expression in different samples, and vertical coordinates represents the statistically significant differences in circRNA expression; **(H)** Sample differences compare up- and downregulated circRNAs.

MicroRNA predicted 643 new microRNAs (**Supplementary Table 1**). The experimental flow was obtained by SmallRNA (**Figure 2A**). Annotation results show that the annotated miRNA in the sample accounts for approximately 12%/18% of all known miRNAs of the species (**Supplementary Table 2**).

### Prediction of miRNA Target Genes and Differentially Expressed microRNA Target Genes

MiRanda software was used to predict possible target sites of the microRNA sequence and the genomic cDNA sequence of the corresponding species. There were 1,048,575 relationships between all miRNAs and their target genes, of which 1059 microRNA were differentially expressed (**Supplementary Table 3**) and of which 24 differentially expressed microRNA genes were predicted and 118,767 target genes were predicted for significantly differentially expressed microRNAs (**Supplementary Table 4**).

### Screening of Differentially Expressed MicroRNAs

Results were screened according to the standard of significant difference (the expression of microRNA changed by more than 2-fold with a P-value < 0.05), and significant up- and downregulation of microRNA expression was monitored. The statistical results showed that the expression of 6 genes in the ck group was significantly higher than that in the ko group, while the expression of 18 genes in the ck group was significantly lower than that in the ko group (**Figures 2B, C**).

The gene IDs with significantly increased expression were as follows: mmu-miR-375-5p; NovelmiRNA-226; NovelmiRNA-362; mmu-miR-7689-5p; NovelmiRNA-288; and NovelmiRNA-239. There was no expression of NovelmiRNA-226; NovelmiRNA-362; NovelmiRNA-288; or NovelmiRNA-239 in the ck group. The ID of genes with significantly decreased expression were as follows: NovelmiRNA-505; mmu-miR-9-5p; mmu-miR-10a-5p; mmu-miR-196b-5p; mmu-miR-152-5p; mmu-miR-467e-5p; mmu-miR-582-5; mmu-miR-139-5p; mmu-miR-125a-5p; NovelmiRNA-403; NovelmiRNA-453; NovelmiRNA-615; NovelmiRNA-131; NovelmiRNA-581; mmu-miR-598-5p; mmu-miR-211-5p; mmu-miR-196a-5p; and mmu-miR-215-5p. Among them, 11 genes, NovelmiRNA-505; mmu-miR-467e-5p; mmu-miR-582-5p; mmu-miR-125a-5p; NovelmiRNA-403; NovelmiRNA-453; NovelmiRNA-615; NovelmiRNA-131; NovelmiRNA-581; mmu-miR-598-5p; and mmu-miR-196a-5p, were not expressed in the ko group.

### CircRNA Statistics and Expression Results

CircRNA analysis process (**Figure 2D**). A total of 2724 circRNAs were predicted, of which the number of known circRNAs was 103. The statistical results of circRNA and its source genes were known (**Supplementary Table 5**). The number of newly discovered circRNAs was 2621, and the number of known circRNAs accounted for 3.78% of the total predicted circRNAs. According to the statistical results of the number of circRNAs in different expression level intervals, in circRNAs with lengths more than 100, the number in the ck group was more than in the ko group. In short circRNAs less than 100, the number in the ko group was more than in the ck group (**Supplementary Table 6**). The statistics of differentially expressed circRNAs (**Supplementary Table 7**) and the distribution map and box diagram of circRNA expression levels under different experimental conditions are shown in (**Figures 2E, F**).

### Screening of Differentially Expressed circRNAs

The results of DESeq2 detection were screened according to the standard of significant difference (differentially expressed circRNAs changed by more than 2-fold with an FDR < 0.05), the significant up- and downregulation of circRNA expression was monitored. The statistical results showed that the expression of 6 genes in the ck group was significantly lower than that in the ko group, while the expression of 10 genes was significantly increased in the ck group compared with the ko group (**Figure 2G, H**).

The IDs of genes with significantly decreased expression are as follows: 1:170787346|170819432; 1:87778922|87881939; 2:93730158|93813964; 10:117694905|117702335; 6:112672124|112681676; and Y:90719917|90805587. The six genes were expressed only in the ko group, not in the ck group.

The gene IDs with significantly increased expression are as follows: 5:30204341|30204750; 11:6193154|6193476; 6:143180587|143186634; 9:72669952|72677531; 13:48932297|48934078; 15:12136396|12140652; 8:84996955|84998291; 7:44915841|44916470; 9:44200960|44281921; and 6:143041352|143061861. These 10 genes were only expressed in the ck group, not in the ko group.

### Cluster Analysis of Differentially Expressed Genes

Cluster analysis calculates the similarity of the data and classifies the data according to the similarity, so that microRNAs (circRNA) with the same function or close relationship can be clustered into groups to identify the function of unknown microRNAs (circRNA) or the unknown function of known microRNAs (circRNA) to infer whether they participate in the same metabolic process or cell pathway together.

**Figure 3:**
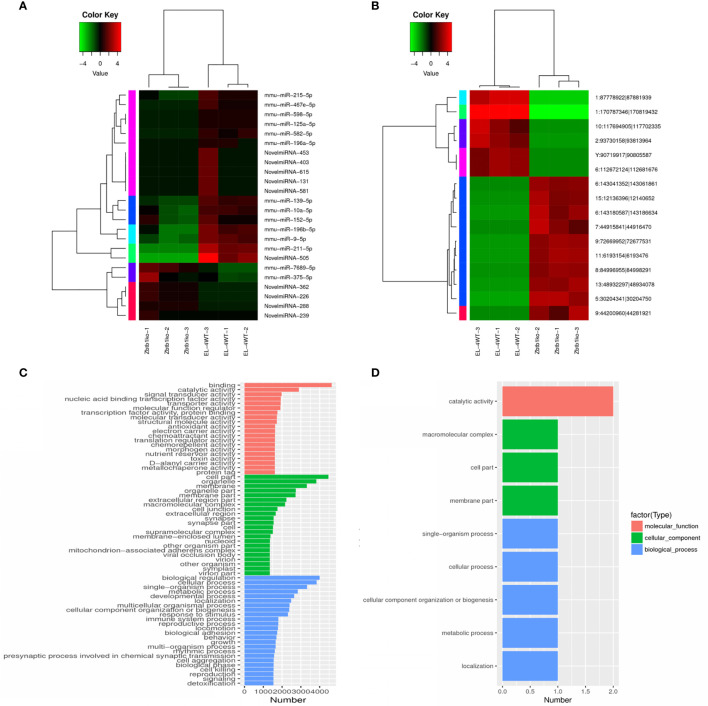
Cluster map of differentially expressed microRNAs and circRNAs according to the log2SRPBM value. Red indicates high expression, and green indicates low expression. The color from green to red indicates higher expression. **(A)** Cluster map of differentially expressed microRNAs; **(B)** Cluster map of differentially expressed circRNAs; GO enrichment histogram. The ordinate is the enriched GO term, and the abscissa is the number of differentially expressed microRNA target genes and circRNA source genes corresponding to the term. Different colors are used to distinguish biological processes, cellular components and molecular functions. **(C)** GO enrichment histogram of differentially expressed microRNA target genes; **(D)** GO enrichment DAG diagram of differentially expressed circRNA source genes in columnar form.

The results of tree clustering in the heat map showed significant differential expression of microRNAs (**Figure 3A**) and circRNAs (**Figure 3B**). Different color regions represent different clustering grouping information, and expression patterns in the same group were similar. The upregulated and downregulated genes had the same function or close relationship and were clustered in the same metabolic or cellular pathway. In addition, the upregulated and downregulated genes may have similar functions or participate in the same biological processes.

### Enrichment GO Term Histogram of Differentially Expressed MicroRNA Target Genes and Differentially Expressed CircRNA Source Genes

The GO enrichment bar chart directly reflects the biological process (BP), cellular component (CC) and molecular function (MF). The distribution of the number of differentially expressed microRNA target genes is shown for the enriched GO terms. We selected the 30 GO terms with the most significant enrichment to show in the diagram, and if there were less than 30, all of them were displayed.

Results showed that in the GO enrichment of microRNA target genes, we enriched the related items of BP, CC and MF. Among them, 30 GO items were concentrated in target genes (p<0.05), as shown in (**Figure 3C**). The items with the most significant activity in molecular biology were binding, catalytic activity and so on. At the same time, a large number of gene products were located in organelles or constituent cells, such as the enrichment of items like cell part, organelle, and membrane. Biological regulation, cellular process, and single-organism process were significantly enriched in biological processes, such as cell growth and maintenance and signal transduction. These results suggest that microRNAs may play an important role in the catalysis of cell metabolism, cell composition and biological regulation.

In the GO enrichment analysis of differentially expressed circRNA source genes, we examined the enrichment of GO terms related to BP, CC and MF. A total of 9 GO terms were significantly enriched (p<0.05), of which the concentrated items were primarily related to cell composition and biological processes (**Figure 3D**), such as macromolecular complex, cell part, membrane part, single-organism process, cellular process, cellular component organization or biogenesis, metabolic process, and localization. A DAG (directed acyclic graph) is the graphical display of the results of GO enrichment analysis of differentially expressed circRNA source genes. DAG diagrams were created for biological process, molecular function and cellular component (**Supplementary Figure 1**). It can be seen that the molecular function-related items enriched in differentially expressed circRNA source genes involve catalytic activity, including ENSMUSG00000027198 (exostosin, glycosyltransferase 2) and ENSMUSG00000030275 (ethanolamine kinase 1). Five genes were enriched in biological process, among which ENSMUSG00000030275 (ethanolamine kinase 1) and ENSMUSG00000020184 (transformed mouse 3T3 cell double minute 2) were enriched in primary metabolic process. Genes enriched in biological process included ENSMUSG00000052566 (hook microtubule tethering protein 2), ENSMUSG00000020184 (MDM2), and ENSMUSG00000030275 (ethanolamine kinase 1). These results suggest that circRNAs may play an important role in glycosylation, acetamide and cholinesterase activity; cytoskeleton composition; and cancer.

### KEGG Enrichment and Scatter Plots of Differentially Expressed MicroRNA Target Genes and CircRNA Source Genes

Based on the KEGG database (http://www.genome.jp/kegg/pathway.html), KEGG annotation and pathway enrichment analysis were performed. In this diagram, the degree of KEGG enrichment was measured by the Rich factor, Q-value and number of genes enriched in a given pathway. The Rich factor refers to the ratio of the number of differentially expressed microRNA target genes located in a given pathway to the total number of annotated genes in that pathway. The larger the Rich factor, the greater the degree of enrichment is. We selected 30 pathway items with the most significant enrichment to display in the map(Qvalue<0.01), and all of them were displayed if the enriched pathway items were less than 30.

**Figure 4:**
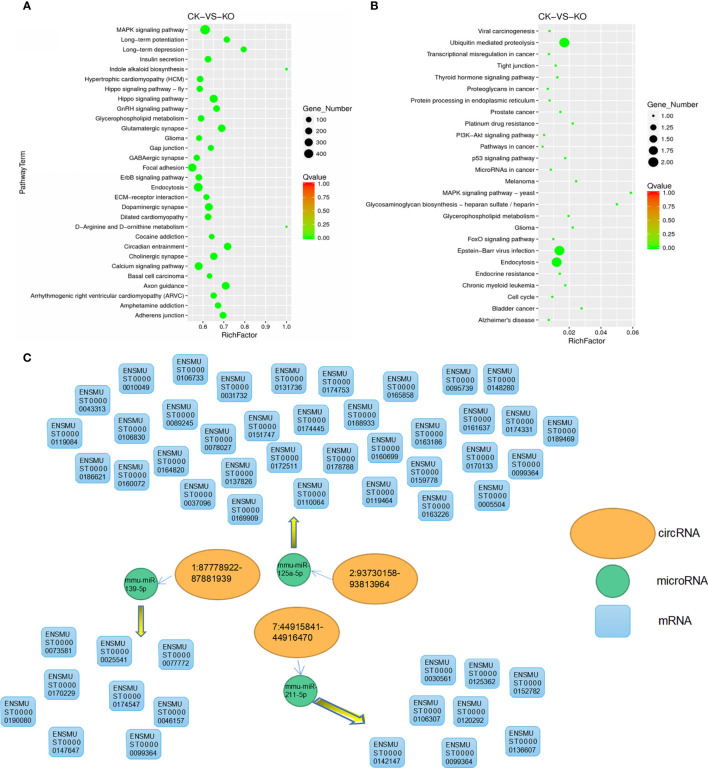
KEGG enrichment dot map. The vertical axis represents the pathway name and the horizontal axis indicates the size of the Rich factor. Dots indicate the number of differentially expressed genes in the pathway, and the color of the dots corresponds to different Q-values. **(A)** Dot plot of KEGG enrichment of differentially expressed microRNA target genes; **(B)** Dot plot of KEGG enrichment of differentially expressed circRNA source genes; **(C)** Regulatory relationship among microRNA, circrna and mRNA.

Results showed that there was a regulatory relationship between mRNA and microRNA in a total of 17,443 mRNAs. The KEGG classification of target genes showed that there were 7014 microRNA target genes involved in the enrichment of 115 pathways (**Supplementary Table 8**), 30 of which showed the most significant enrichment (**Figure 4A**). The KEGG enrichment results of circRNA(**Supplementary Table 9**) source genes show that there are 26 pathways that are significantly enriched (**Figure 4B**), and a total of 5 target genes were enriched in the pathways. Among them, pathways related to cancer, tumor and ubiquitin were significantly enriched, such as ubiquitin-mediated proteolysis, viral carcinogenesis, and transcriptional dysregulation in cancer. It also proves the regulatory function of the Zbtb1 gene in cancer, tumor and ubiquitin-related pathways.

Among them, the signaling pathway of coenrichment of microRNA target gene and circRNA source gene is metabolism glioma, MAPK signaling pathway, glycerophospholipid, and endocytosis. It may indicate that these four pathways are regulated by differentially expressed microRNA target genes and circRNA source genes to a certain extent.

### MiRNA-circRNA-mRNA Association Analysis

One of the important functions of circRNA in different species is that it can function as an miRNA sponge (ceRNA) that can competitively binds to miRNA to regulate the expression of target genes. For animal samples and plant samples, we used miRanda and psRobot software to predict the targeting relationship between circRNA、miRNA and mRNA, and we ultimately obtained results on the regulation of the following three pairs as shown in **Figure 4C**.

## Discussion

In this experiment, we successfully constructed an EL4 cell line with Zbtb1 gene knockout by the CRISPR-Cas9 technique and screened out monoclonal cells. The results showed that the subcutaneous growth rate of EL4 cells with Zbtb1 gene knockout in C57BL/6 mice was significantly slower than that of wild type EL4 cells.

The results of microRNA expression analysis showed that the expression of 18 genes in the ck group was significantly lower than that in the ko group, while the expression of 6 genes was significantly increased in the ck group compared with the ko group. By analyzing the differential expression of circRNAs between the ck and ko groups, it was found that 6 genes were significantly decreased, and 10 genes were significantly increased in the ck group compared to the ko group. Through GO enrichment analysis of differentially expressed microRNA target genes and circRNA source genes and KEGG enrichment analysis, it is speculated that microRNA target genes play an important role in the catalysis of cell metabolism, cell composition and biological regulation. CircRNA-derived genes may play an important role in glycosylation, acetamide and cholinesterase activity; cytoskeleton composition; and cancer and participate in the regulation of cancer-, tumor- and ubiquitin-related pathways. Through correlation analysis, results of the regulation relationship between three pairs of microRNA and circRNA were obtained.

The key to studying the biological function and mechanism of microRNAs is to accurately identify target genes of the microRNA. By binding to RISC and acting on the 3’UTR of its target genes, microRNA degrades its target genes or inhibits the translation of its target genes, participating in a wide variety of biological processes, such as cell proliferation, differentiation, development and apoptosis, and has an important impact on a variety of diseases. MicroRNA is abnormally expressed in a variety of tumors, which has important reference values clinically (Stadthagen et al., 2013).

Of the 18 microRNAs decreased in the ck group compared to the ko group, 6 were not annotated. Among the 12 annotated genes, mmu-miR-9-5p was upregulated in mouse gastric cancer cells, lung cancer cells and head and neck squamous cell carcinoma and targets NF- kappa B and regulates the growth of gastric cancer cells; (Liu and Kohane, 2009; Wan et al., 2010; Yu et al., 2012; Christensen et al., 2014); mmu-mir-10a is highly expressed in a variety of tumor cells and can be used as an effective diagnostic marker for colorectal cancer (Hoesel and Schmid, 2013); mmu-miR-196b-5p promotes the growth of bladder tumor cells, glioma cells and chronic myeloid leukemia cells, inhibits the metastasis of breast cancer cells and inhibits the growth of liver cancer cells and colon cancer cells (Guan et al., 2010; Li Y. et al., 2010; Rebucci et al., 2015; Jiang et al., 2017; Zhu et al., 2017); mmu-miR-152-5p inhibits the growth of ovarian and gastric cancer cells and is downregulated in prostate cancer, neuroblastoma, melanoma cells and intestinal cancer cells, while mmu-miR-582-5p inhibits the proliferation of bladder cancer cells (Haflidadóttir et al., 2010; Song et al., 2011; Terrile et al., 2011; Spencer et al., 2012; Wang et al., 2016; Lin et al., 2018); microRNA-139-5p inhibits the proliferation of colorectal cancer and hepatocellular carcinoma cells and is downregulated in glioblastoma and breast cancer cells (Silber et al., 2008; Wong et al., 2011; Biagioni et al., 2012; Zhang et al., 2014); mmu-miR-196a-5p promotes breast tumor, esophageal cancer, glioma cells and colorectal cancer, and its expression is upregulated in gastrointestinal stromal tumor, breast cancer and pancreatic cancer tissues and cells (Luthra et al., 2008; Hoffman et al., 2009; Hui et al., 2009; Schimanski et al., 2009; Zhang et al., 2009; Niinuma et al., 2012); mmu-miR-125a inhibits the proliferation of hepatocellular carcinoma, laryngeal squamous cell carcinoma and endometrial cancer cells (Bi et al., 2012; (Liang et al., 2010); Jiang, 2011; Sun et al., 2017); miR-598 inhibits metastasis of colorectal cancer (Jia et al., 2017); mmu-miR-211-5p has inhibitory effects on colorectal cancer and melanoma (Peter et al., 2012); mmu-miR-215-5p inhibits the proliferation of colorectal tumor and epithelial ovarian cancer (Lin et al., 2017; Vychytilova-Faltejskova et al., 2017). Of the 6 microRNAs increased in the ck group compared to the ko group, 4 did not have available information. Expression of mmu-miR-375 is upregulated in prostate cancer and hepatocellular carcinoma and decreased in squamous cell carcinoma. It inhibits the proliferation of gastric cancer cells and inhibits the metastasis of colorectal cancer (Hui et al., 2010; Li L. et al., 2010; Tsukamoto et al., 2010; Selth et al., 2012; Xu et al., 2016). From a large number of research results, most of the differentially expressed microRNA between the ck and ko groups inhibit cancer cell proliferation and metastasis, which is consistent with the experimental results that the cell growth of the ko group was slower than the ck group.

Due to the large amount of microRNA production and extensive regulatory effects, the functional results of enrichment of microRNA target gene GO are very complex. There are many enrichment results in biological process, cellular component and molecular function, and 115 pathways were significantly enriched in KEGG enrichment analysis, in which micro target genes accounted for the largest proportion of regulation in indole alkaloid biosynthesis and D-arginine and D-ornithine metabolism. The largest number of target genes were enriched in MAPK signaling pathway, focal adhesion and endocytosis.

CircRNAs can cis-regulate the expression of parental genes. On the one hand, circRNAs can interact with RNA-binding proteins to affect the expression of parental gene mRNA; on the other hand, competitive complementary pairing between introns during the formation of cyclic RNAs can reach a balance with linear RNA, affecting mRNA expression and even protein translation. CircRNAs can also play the role of competitive endogenous RNA-ceRNA adsorption of microRNA. Due to the stability of cyclic RNA, the potential adsorption capacity of microRNA in the body is stronger than linear either mRNA or lncRNA. Recent studies have shown that some cytoplasmic circRNAs can be effectively translated into detectable peptides, highlighting the importance of circRNAs in cellular physiological functions. Cap-independent translation initiation mediated by internal ribosome entry sites (IRES) and N-methyladenosine (MA) is considered to be a potential mechanism of circRNA translation. So far, several translated circRNAs have been found to play a key role in human cancer (Lei et al., 2020; Wu et al., 2020).

The results of GO enrichment analysis of circRNA showed that the concentrated enrichment items were primarily related to cell composition and biological processes and may play an important role in glycosylation, acetamide and cholinesterase activity; cytoskeleton composition; and cancer. The results of KEGG pathway analysis of circRNA-derived genes showed that pathways related to cancer, tumor and ubiquitin were significant. Four pathways were enriched in both microRNA target genes and circRNA source genes.

In many gastrointestinal tumors, the regulation of circRNAs is disrupted and is related to metastasis and invasion. There are differences in the function and expression of some circRNAs in gastrointestinal tumors, such as gastric cancer, colorectal cancer, esophageal cancer, hepatocellular carcinoma, gallbladder cancer and pancreatic cancer, and they can be used as biomarkers. CircRNAs can be used as prognostic markers and targets for the development of new therapeutic methods (Petitdemange et al., 2019; Naeli et al., 2020). Some studies have shown the potential of targeting circRNAs as an important strategy for regulating TME, overcoming cancer drug resistance and improving treatment outcomes (Zhang Q. et al., 2020).

As research on circRNAs has only appeared in recent years, their regulatory role in the body is not understood in large part. Therefore, the study of circRNA still has a long way to go.

## Data Availability Statement

The datasets presented in this study can be found in online repositories. The names of the repository/repositories and accession number(s) can be found below: The raw data for this article were deposited in the National Center for Biotechnology Information (NCBI) Sequence Read Archive (SRA) database under BioProject number PRJNA 672845; PRJNA673569.

## Ethics Statement

All experiment protocols were approved by the Institutional Animal Care and Use Committee of Jilin Agricultural University.

## Author Contributions

XC, J-HW, and C-FW conceived and designed research. C-WS and Y-YL conducted experiments. J-HW analyzed data. J-HW and C-WS wrote the manuscript. YZ, M-YC, R-YW, YS, Y-LJ, W-TY, D-DZ, H-BH, L-PY, and G-LY contributed to the work. All authors contributed to the article and approved the submitted version.

## Funding

This work was supported by the National Key Research and Development Program of China (2017YFD0501000, 2017YFD0500400), the National Natural Science Foundation of China (31672528, 31700763, 81760287, 31941018, 32072897) and the Science and Technology Development Program of Jilin Province (20180201040NY, 20190301042NY, 20200402041NC), Science and Technology Project of the Education Department of Jilin Province during the 13th Five-year Plan (JJKH20200360KJ).

## Conflict of Interest

The authors declare that the research was conducted in the absence of any commercial or financial relationships that could be construed as a potential conflict of interest.

## Supplementary Material

Supplementary Figure 1 The branches of DAG diagrams represent inclusion relationships, and the functional range defined from top to bottom becomes smaller and smaller. The top 10 results of GO enrichment analysis are generally selected as the primary nodes of directed acyclic graphs, and the associated GO terms are displayed together through inclusion relationships. Biological process and DAG diagram.

Supplementary Table 1 Predicted list of new microRNAs.

Supplementary Table 2 (1) Sample: Sample name; (2) Num_hit_miRNA: Number of miRNA commented to; (3) All_miRNA: All known miRNA numbers of this species; (4) Hit_miRNA/All_miRNA: Percentage of miRNA annotated.

Supplementary Table 3 Differentially expressed microRNA list. (1) Average normalization of the number of Id: microRNA ID; (2) baseMean: fragments, the average value under the two conditions; (3) log2FoldChange: takes log; (4) lfcSE: as the base to estimate the standard error of log2FoldChange; (5) stat: Wald test; (6) pval: significant difference significant value; (7) padj: FDR-corrected P-value.

Supplementary Table 4 Differentially expressed miRNAs and their target genes.

Supplementary Table 5 CircRNA list commented to (1) circRNA_ID: circRNA number (2) strand: plus or minus chain (3) annotated_circRNA_site: known circRNA location information (4) annotated_circRNA_ID: known circRNA ID.

Supplementary Table 6 Statistical tables of circRNA quantity between different expression levels.

Supplementary Table 7 (1) Id: circRNA ID; (2) baseMean: fragments is normalized, and the average value under the two conditions; (3) log2FoldChange: takes log (4) lfcSE: as the base to estimate the standard error of log2FoldChange; (5) stat: Wald test (6) pval: significant difference significant value; (7) padj: FDR-corrected P-value.

Supplementary Table 8 (1) Pathway of microRNA target genes: pathway of microRNA target gene ID; (2) Pathway: pathway name; (3) DEGs with pathway annotation: the number of genes in the differential analysis comparison group; (4) All genes with pathway annotation: the number of genes in all gene backgrounds; (5) P-value: this pathway has an increased P-value; (6) Q-value: this pathway has an increased Q-value; (7) Genelist: this pathway is enriched in differentially expressed genes; (8) KOlist: this pathway is enriched in differentially expressed KO genes.

Supplementary Table 9 Pathway of circRNA-derived genes. (1) PathwayID: pathway ID; (2) Pathway: pathway name of circRNA source genes; (3) DEGs with pathway annotation: number of genes in the difference analysis comparison group; (4) All genes with pathway annotation: the number of genes in all gene backgrounds; (5) P-value: this pathway has an increased P-value; (6) Q-value: this pathway has an increased Q-value; (7) Genelist: this pathway is enriched in differentially expressed genes; (8) KOlist: this pathway is enriched in differentially expressed KO genes.
