# Supplementary figures and images for: MicroRNA and circRNA Expression Analysis in a Zbtb1 Gene Knockout Monoclonal EL4 Cell Line
Source: Front Cell Infect Microbiol. 2021 Jul 5;11:706919. doi: 10.3389/fcimb.2021.706919 (PMC8287301; doi:10.3389/fcimb.2021.706919)

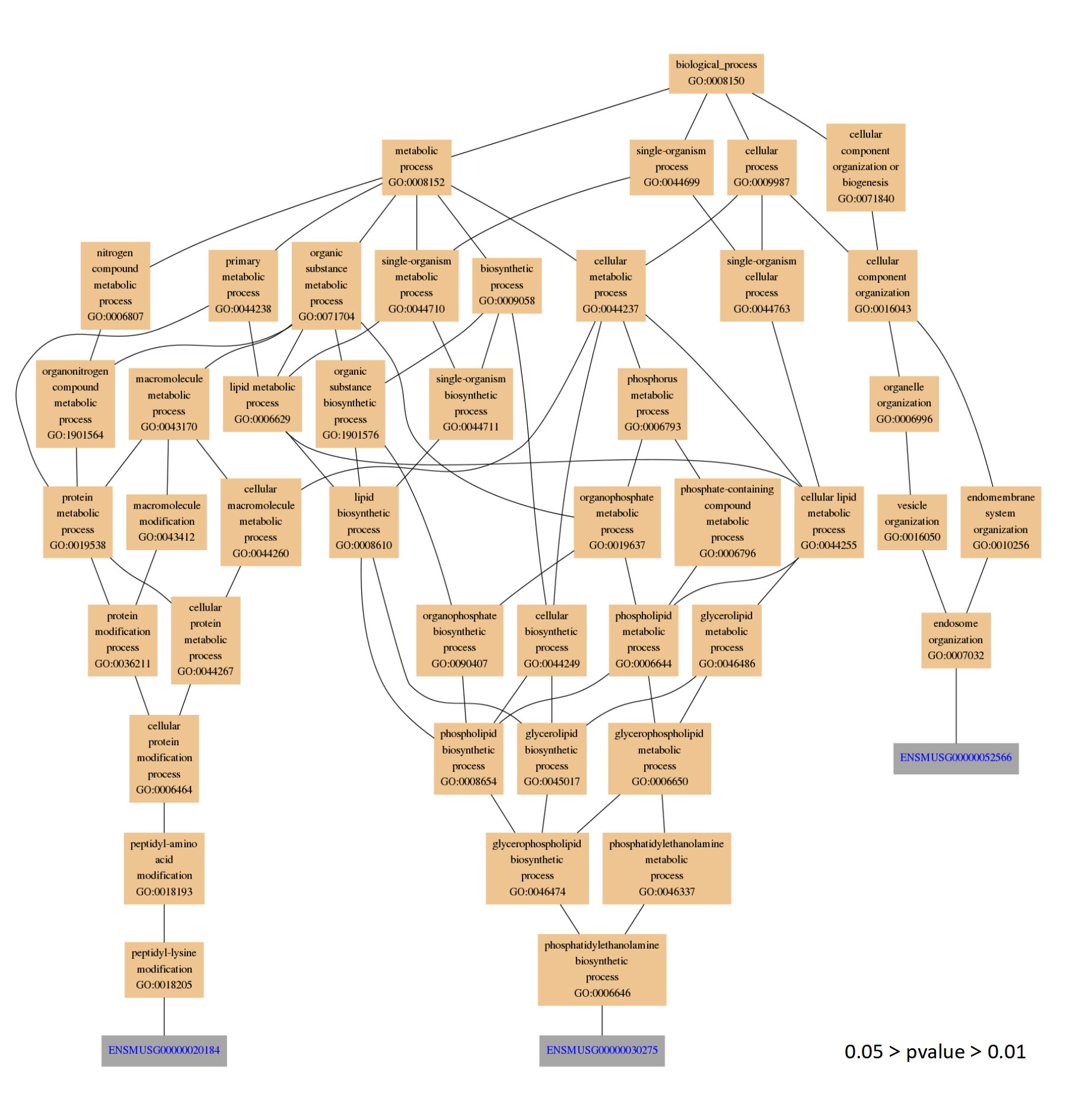

Supplement: Supplementary Figure 1 — The branches of DAG diagrams represent inclusion relationships, and the functional range defined from top to bottom becomes smaller and smaller. The top 10 results of GO enrichment analysis are generally selected as the primary nodes of directed acyclic graphs, and the associated GO terms are displayed together through inclusion relationships. Biological process and DAG diagram. [file Image_1.tif]
